# Development of multi-level standards of care recommendations for sickle cell disease: Experience from SickleInAfrica

**DOI:** 10.3389/fgene.2022.1052179

**Published:** 2023-01-12

**Authors:** Vivian Paintsil, Mwashungi Ally, Hezekiah Isa, Kofi A. Anie, Josephine Mgaya, Malula Nkanyemka, Victoria Nembaware, Yaa Gyamfua Oppong-Mensah, Flora Ndobho, Lulu Chirande, Abel Makubi, Obiageli Nnodu, Ambroise Wonkam, Julie Makani, Kwaku Ohene-Frempong

**Affiliations:** ^1^ Department of Child Health, School of Medicine and Dentistry, Kwame Nkrumah University of Science and Technology, Kumasi, Ghana; ^2^ Directorate of Child Health, Komfo Anokye Teaching Hospital, Kumasi, Ghana; ^3^ Sickle Cell Programme, Muhimbili University of Health and Allied Sciences (MUHAS), Dar es Salaam, Tanzania; ^4^ Department of Haematology and Blood Transfusion, Centre of Excellence for Sickle Cell Disease Research and Training (CESRTA), University of Abuja, Abuja, Nigeria; ^5^ London North West University Healthcare NHS Trust, Imperial College London, London, England; ^6^ Division of Human Genetics, Faculty of Health Sciences, University of Cape Town, Cape Town, South Africa; ^7^ Sickle Cell Foundation of Ghana, Accra, Ghana

**Keywords:** standards of care, Sickle Cell Disease, SickleInAfrica, Sickle Pan African Research Consortium (SPARCO), Sub-Saharan Africa

## Abstract

**Introduction:** Sickle Cell Disease (SCD) causes significant morbidity and mortality particularly in sub-Saharan Africa (SSA) where it contributes to early childhood deaths. There is need to standardize treatment guidelines to help improve overall SCD patient health outcomes. We set out to review existing guidelines on SCD and to set minimum standards for management of SCD for the different referral levels of healthcare.

**Methods:** A standards of care working group (SoC-WG) was established to develop the SoC recommendations. About 15 available SCD management guidelines and protocols were reviewed and themes extracted from them. The first draft was on chosen themes with 64 major headings and subtopics. Using a summarised WHO levels of referral document, we were able to get six different referral levels of healthcare. The highest referral level was the tertiary facilities whilst the lowest level was the home setting. Recommendations for SCD management for the regional, district, sub-districts, health posts and CHPs compounds were also drafted.

**Results:** The results from this review yielded a guidelines document which had recommendations for management of SCD on 64 topics and subtopic for all the six (6) different referral levels.

**Discussions:** Every child with SCD need to receive comprehensive care that is coordinated at each level. This recommendation is unique in terms of the availability of recommendations for different levels of care as compared to the traditional guidelines which is more focused at the tertiary levels. Patients can access care at any of the other lower referral hospitals and be managed with recommendations that are in keeping with institutional resources at that level. When such patients need care that requires expertise that is not available at that level, the recommendations will be to refer to the appropriate referral level where those expertise are available. This encourages patients to have good clinical care nearer their homes but also having access to specialist screening modalities and expertise at the tertiary hospitals if need be. With this, patient are not limited to a specific referral level when interventions cannot be instituted for them.

**Conclusion:** This SoC recommendations document is a useful material that can be used for consistent standards of treatment in SSA.

## Introduction

Sickle Cell Disease (SCD) is a group of inherited red blood cell disorders characterized by presence of abnormal haemoglobin resulting in formation of hard and sticky C-shaped red cells (CDC, 2022). This is caused by a single base-pair point mutation in the 6^th^ position of the beta globin chain leading to substitution of the amino acid glutamic acid to valine (GAG to GTG) (Genetic Mutation, 2022). The most common types of SCD are SCD-SS, SCD-SC and SCD-S beta thalassemia. SCD causes significant morbidity and mortality particularly in SSA. SCD is one of the leading causes of hospitalization and mortality in under-fives in Africa. The clinical manifestation of SCD are varied and due to the short life span of sickled red blood cells and their tendency to get stuck in the blood vessels and impair the blood flow. Symptoms and signs include anaemia, pain episodes, infections, stroke, and other symptoms depending on the organ involved.

Over 300,000 babies are born annually worldwide with SCD and most of them are found in low and middle income countries (LMICs). About 75% or more of these births are in Sub-Saharan Africa (SSA) (Piel et al., 2013). SCD contributes to the high health burden in SSA and to early childhood deaths if no interventions are put in place. If existing evidence-based cost-effective interventions were implemented in SSA, they could prevent about 70% of these childhood deaths. Therefore, there is need to implement strategies and treatment guidelines to help improve overall SCD patient health outcomes through standardization of quality care. A commonly used approach is the use of “Standard of Care” guidelines for healthcare professionals.

In September 2015, the National Heart, Lung and Blood Institute (NHLBI) of the National Institute of Health (NIH) of the United States of America issued a Request for Application (RFA-HL-17-006) for establishment of a “Sickle Cell Disease in Sub-Saharan Africa: Collaborative Consortium”. A multinational collaboration among Muhimbili University of Health and Allied Sciences (MUHAS), Dar es Salaam, Tanzania as hub and three sites; MUHAS, Dar es Salaam, Tanzania, University of Abuja, Abuja, Nigeria and The Kwame Nkrumah University of Science and Technology, Kumasi, Ghana applied for and was awarded the grant (U24HL135881) (Yoshizawa et al., 2019).

Sickle Pan Africa Research Consortium (SPARCo) was established with a vision to transform the health of individuals with SCD in SSA through the establishment of a robust and portable framework for research and healthcare. Its first goal was to reduce morbidity and mortality in SCD in Africa through implementation research that will demonstrate the feasibility of introducing newborn screening (NBS) and providing comprehensive care to prevent, identify, treat and manage acute and chronic complications. Specific aim two of the proposal was to develop, implement and evaluate a resource-based, multi-level “Guidelines for Management of SCD in SSA”. The specific objectives were as follows;

1. To review existing guidelines on SCD
2. To set minimum standards for management of SCD based on institutional and human resources
3. To field test the compiled SoC guidelines in the various countries (Makani et al., 2020).

### Standard of care definition

The term “standard of care” (SoC) refers to the reasonable degree of care a person should provide to another person, typically in a professional or medical setting (Standard of Care, 2022). Medically, it refers to treatment guidelines that are accepted in the medical community as the most appropriate for the treatment of a certain disease or condition (NCI, 2011). SoC provide clinical practice guidelines which outline formal diagnostic and treatment processes that a doctor will follow in management of a patient with a specific illness. SoC are commonly developed by a medical society or organization working on the specific disease. The use of SoC has been found to improve quality of care for patients and reduce incidences of malpractice (Mccabe et al., 2009).

### Importance of SoC tailored for specific levels of healthcare facilities

There are various SCD SoC guidelines worldwide (Heart and Institute, 2014; De Montalembert et al., 2011; Sicklecell Society, 2018; Wilson, 2019; MEDBOX, 2022). However, the majority of the existing guidelines are one level guidelines, developed to be used in tertiary level healthcare facilities where resources and expertise are available (Alli et al., 2014; Heart and Institute, 2014; Sicklecell Society, 2018). This is not feasible in many African settings where there are limited tertiary level healthcare facilities (see Table 1 for descriptions of classification of the different levels of healthcare facilities). Furthermore, some tertiary level healthcare facilities in Africa are not well-resourced, which results in some well-established SCD centres having to adapt existing SCD SoC based on the availability of resources. Given that anecdotal data shows that majority of patients are seen and managed at the lower level healthcare facilities before being referred to tertiary hospitals, the use of the tertiary level guidelines by the lower level healthcare facilities would be inappropriate. Although there are efforts to develop guidelines at the institutional and national levels, we decided to develop multiple recommendations for SoC guidelines to match the availability of facilities and expertise for different levels of healthcare facilities in SSA (Table 1).

**TABLE 1:** Summary of WHO classification of healthcare facility levels.

Standard version	Last referral hospital (LRH)	University teaching hospital (private, government or mission); may serve as a regional centre for sickle cell Disease	Highest level of medical care available in the country or region
Limited Version 1	Secondary Referral Hospital (SRH)	Regional Hospital (private, government or mission), Community Hospital; may serve as a Regional Centre for Sickle Cell Disease	Multi-specialist intramural and extramural care and services. May also have special expertise in some particular diagnostic or treatment domain that may qualify it as the last referral for that specific subject
Limited Version 2	First Referral Hospital (FRH)	District Hospital (private, government or mission); may serve as a Sub-Regional Centre for Sickle Cell Disease	Intramural medical care of a level or complexity beyond that feasible by ambulatory care in the particular area, district, or region
Limited Version 3	Level II Health Centre	Health Centre	Ambulatory and curative services; health promotion, prevention, and education; support for Level I Health Centres; maternity and observation beds; outpatient operating room; staffed by multidisciplinary team of professional and auxiliary health workers; population served maximum 100,000
Limited Version 4	Level I Health Centre (HC I)	Dispensary, Health Post, Health Sub-center, or Community-based Health Planning and Services (CHPS) Compound (Ghana)	Limited ambulatory and curative services; community development; no beds—possibly one maternity bed; staffed by auxiliary nurse midwife; population served <10,000
Home Version	Home	Home (urban or rural)	No medical expertise; with or without electricity or refrigeration; with or without private transportation

The Sickle Cell Foundation of Ghana in 2012 were the first to develop SCD guidelines for each of the different referral levels of healthcare for Ghana. The current work adopted this multi-referral level guideline development. The Ghana guidelines recognises the disparities in the availability of resources at the different levels of healthcare facilities and incorporates it into the guidelines. These levels of healthcare was adopted from the WHO document on the levels of referral. This WHO document has five referral levels for healthcare facilities depending on resources available. The highest level of medical care available in the country comprise the tertiary level and mostly a University or teaching hospital. There was the inclusion of a 6^th^ referral level which is the home SCD management guidelines targeted at the patient or their parents. The highest referral level is the tertiary level health facility. There are recommendations for the regional level facilities, district level facilities, health centres, health posts/dispensaries/Community-based health planning and services (CHPs) compound which is unique to Ghana and finally the home level recommendations. The highest level guideline is also denoted as the “Standard Version” for the tertiary healthcare facilities. This standard version is then also scaled down for the other lower-level health facilities. These are also denoted as limited Version 1 for the Regional hospitals, Limited Version 2 for the district hospitals, limited version 3 for the health centres and limited version 4 for the dispensary, health post or CHPs compounds. The Sickle Cell Foundation of Ghana has a unique guideline, the home management guideline as the last level.

The Sickle Pan Africa Research Consortium (SPARCO) adopted the SoC guidelines design approach developed by the Sickle Cell Foundation of Ghana. This design is especially suitable for developing countries where healthcare infrastructure and human resources are largely low with patients being managed with restricted resources. It is our expectation that the SPARCO SCD SoC recommendations will guide all cadres of healthcare providers at all the different referral levels of healthcare, therefore improving the quality of care given to individuals with SCD.

## Methodology

### Setting up of the working group

A standards of care working group (SoC-WG) was established to develop the SoC recommendations for use in the various referral levels of healthcare. The SoC-WG comprised of seven members who were elected from all the three sites (Ghana, Nigeria and Tanzania) in the Sickle Pan Africa Research Consortium (SPARCo). Each of the sites nominated two (2) doctors with a background in haematology to be part of the team. One member of the group who was a clinical psychologist, although not part of SPARCO, was co-opted to help with the section on psychosocial support for Sickle Cell Disease (SCD) patients as he had lots of experience with it. The chairperson of the working group was a Professor of Haematology with experience in guidelines development and the other members of the group were people who had helped with guidelines at their national levels.

### Project management

A detailed work plan (Table 2) was developed according to the grant proposal and members worked according to the fixed timelines. To achieve our goals, assignments were initially discussed and distributed to members. Monthly online meetings were held and communications *via* email and WhatsApp were conducted to keep track of the work. In-person working meetings were also held during the consortium’s bi-annual meetings.

**TABLE 2:** SPARCO standards of care working group: Detailed work plan.

Yr	#	Activities	Methods	Start date	End date	Responsible	Status
1	1	Set up a Care Standards Committee within SPARCO health working group					
	2	Collect and review all the SCD management guidelines and protocols available at Consortium sites					
	3	Review the latest versions of management guidelines from the US, UK, France, Brazil and Jamaica					
2	4	From reviews: develop “Common” Guidelines for Management of SCD in SSA					
	5	Set minimum standards for management of SCD based on institutional technical and human capacity for each level of healthcare					
3	6	One-year of extended field testing, including SPAN sites					
	7	Final versions of minimum standards, in English, French and Portuguese, will be disseminated mostly through the internet, hard copies in areas where internet service is poor and a Mobile application (App) version					
4	8	Set approval of the Regionalized Common guidelines from Ministry of Health, Medical and Nursing organizations, and the major healthcare institutions within the regions for incorporation into their system of implementation					
	9	A plan for continual review and modification of the Final Versions of the Regionalized Guidelines of Management of SCD in SSA will be developed					

SPARCO, Sickle Pan African Research Consortium; SCD, Sickle Cell Disease; US, United States; UK, United Kingdom; SSA, Sub-Saharan Africa

A database detailing the elements of the SoC recommendations with the activities to be conducted was developed by the members of the working group. These elements were agreed upon by consensus after appraisal of the literature available. This database for the review process included the contact information for members of the SoC WG, the workplan and activity tracker for the working group members. Metrics to analyse and compare guidelines were also developed. The first task was to review all the available SCD management guidelines and protocols.

### SCD guidelines review

Before the review process, several stakeholders in and outside Africa were contacted for a copy of their SCD management guidelines. The SoC WG members reviewed 15 guidelines from US, UK, France, Brazil and Jamaica and from the three sites in SPARCo and SADaCC. These guidelines were grouped into African (Alli et al., 2014; MEDBOX, 2022) and non-African guidelines (Sickle Cell Unit, 1997; Ali Susanna, 2008; Sicklecell Society, 2010; De Montalembert et al., 2011; NICE, 2012; CanHaem, 2014; Heart and Institute, 2014; Colombatti and Sainati, 2016; Sicklecell Society, 2018; Sirigaddi et al., 2018; Liem et al., 2019; Wilson, 2019). These guidelines were mostly national guidelines for the tertiary level healthcare (Table 3).

**TABLE 3:** Existing guidelines reviewed by the SoC working group.

African Scd General Guidelines	Non-African Scd General Guidelines
Management of Sickle Cell Disease- The United Republic of Tanzania	Management of children with Sickle Cell Disease in Europe: current situation and future perspectives. R Colombatti and L. Sainati (16)
National Guidelines for the Control and Management of Sickle Cell Disease—Federal Ministry of Health, Nigeria (10)	Sickle Cell Disease: The Clinical Care Guidelines of the Sickle Cell Unit– Watermarked Jamaica. (18)
Recommendations for the management of sickle cell disease in South Africa. Alli N.A. et al. (14)	Consensus statement on the Care of Patients with Sickle Cell Disease in Canada, CanHaem (15)
Draft Sickle Cell Management Guidelines for Ghana	• NHLBI- Evidence based management of Sickle Cell Disease—Expert Panel report 2014 (9)
	• A parent’s guide to managing Sickle Cell Disease. NHS(UK) - Screening Programme- Sickle Cell and Thalassaemia (19)
	• Sickle Cell Disease in childhood. Standards and guidelines for clinical care NHS (UK)- 2010 (24)
	• ENERCA Clinical recommendations for disease management and prevention of complications of sickle cell disease in children. Mariane de Montalembert, et al. (23)
	• Standards for the clinical care of adults with Sickle Cell Disease in UK. Sickle Cell Society, 2008 (20)
	• Outcomes of febrile events in pediatric patients with sickle cell anaemia. Krishnaveni Sirigaddi, Inmaculada Aban, Amelia Jantz et al. (22)
	• Standards for the clinical care of adults with Sickle Cell Disease in UK. Sickle Cell Society, 2018 (12)
	• Sickle cell acute painful episode; Management of an acute painful sickle cell episode in hospital (NICE) 2012 (21)

### Appraisal of guidelines

The working group members were assigned sets of guidelines from the different countries other than their own country to review. They were assigned the guidelines based on their specialties and area of interests making sure that assignments were evenly distributed. From this review, sets of themes which were common in all the guidelines were documented and other themes which were deemed important but not common in all the guidelines were also extracted and agreed upon by the group. These themes were compiled into a single document and afterwards, an in-depth review was undertaken from which the detailed guidelines were written.

The themes that were chosen were grouped into the following major headings (Table 4): Diagnosis of sickle cell disease and related conditions, Health maintenance and preventive therapy, Screening for specific complications of SCD, management of acute complications of SCD, management of chronic complications of SCD and special management protocols e.g., hydration guide, analgesic therapy and their use in SCD and hydroxyurea therapy. Under these major headings were other sub topics for consideration. These sections made up 64 topics and subtopics.

**TABLE 4:** Major themes/topics and subtopics identified.

S/NO	Theme/Topic	Subtopics
1	Diagnosis of Sickle Cell Disease (SCD) and Related Conditions	A. Tests for hemoglobin (Hb) type using Hb separation or specific immunologic methods, and DNA-based mutation analyses for confirmation
		B. Tests to diagnose heterozygous beta thalassemia (trait) in those with “No Hb variant” visible by the Hb separation methods (Hb phenotypes FA, AF, AFA2, AA2F, AA2, A)
		C. Rapid Screening and Point of Care tests for Hb S, C, A
		D. DNA- based tests
2	Health Maintenance and Preventive Therapy	A. Organizing Clinical Care (Outpatient routine; outpatient acute; and inpatient)
		B. Infection Prevention: General
		C. Immunization
		D. Prevention of invasive pneumococcal disease (IPD)
		E. Prevention of Malaria
		F. Prevention of enteric Gram-negative organisms (*salmonella*, *E. coli*, *klebsiella*, etc)
		G. Genetic and Reproductive counselling
		H. Female reproductive health (Pregnancy, Contraception and Fertility)
		I. Male reproductive health
		J. Nutrition
		K. Growth and development monitoring in children
		L. Education and psychosocial counselling
		M. Organizing support groups
		N. Transitioning of adolescents to adult care
		O. Travel management
3	Screening for Specific Complications of Sickle Cell Disease	A. Screening for stroke risk
		B. Screening for renal disease
		C. Screening for retinopathy
		D. Screening for pulmonary disease
		E. Screening for cardiovascular disease
		F. Screening for hypertension
		G. Screening for red cell antibodies related to pregnancy
4	Management of Acute Complications of SCD	A. Acute anaemia
		B. Acute chest syndrome
		C. Acute hepatobiliary complications
		D. Acute ocular complications
		E. Acute renal failure
		F. Acute SCD pain (vaso-occulsive pain episode, VOPE, or “pain crisis)
		G. Acute splenic sequestration
		H. Acute stroke
		I. Fever and other signs of infection
		J. Osteomyelitis and septic arthritis
		K. Multisystem Organ Failure
		L. Priapism
5	Management of Chronic Complications of Sickle Cell Disease	A. Avascular necrosis
		B. Cardiac complications
		C. Chronic hypersplenism
		D. Chronic pain
		E. Endocrine complications
		F. Gastrointestinal complications
		G. Leg ulcers
		H. Nocturnal enuresis
		I. Ophthalmologic complications
		J. Psychological complications
		K. Renal complications
		L. Pulmonary complications
		M. Seizures under neurological complications
		N. Stuttering/Recurrent Priapism
6	Special Management Protocols	A. Analgesic Medications and their Use in Sickle Cell Disease
		B. Haematopoietic stem cell transplantation
		C. Hydration guide
		D. Hydroxyurea Therapy in Sickle Cell Disease
		E. Peri-operative care and surgery
		F. Transfusion Therapy in Sickle Cell Disease/Iron chelation
		G. Organizing SCD clinical service (outpatient routine and acute care, in patient)

Specific subtopics were then assigned to working group members to review the different guidelines and adapt them to suit our population. There was the need to develop the guidelines to also suit lower referral health facilities that also manage SCD patients. The working group adopted a classification of healthcare facilities based on the summarised WHO classification to get five referral health facility levels for the recommendations (Table 1).

### Consensus and preparation of final guidelines

**FIGURE 1:**
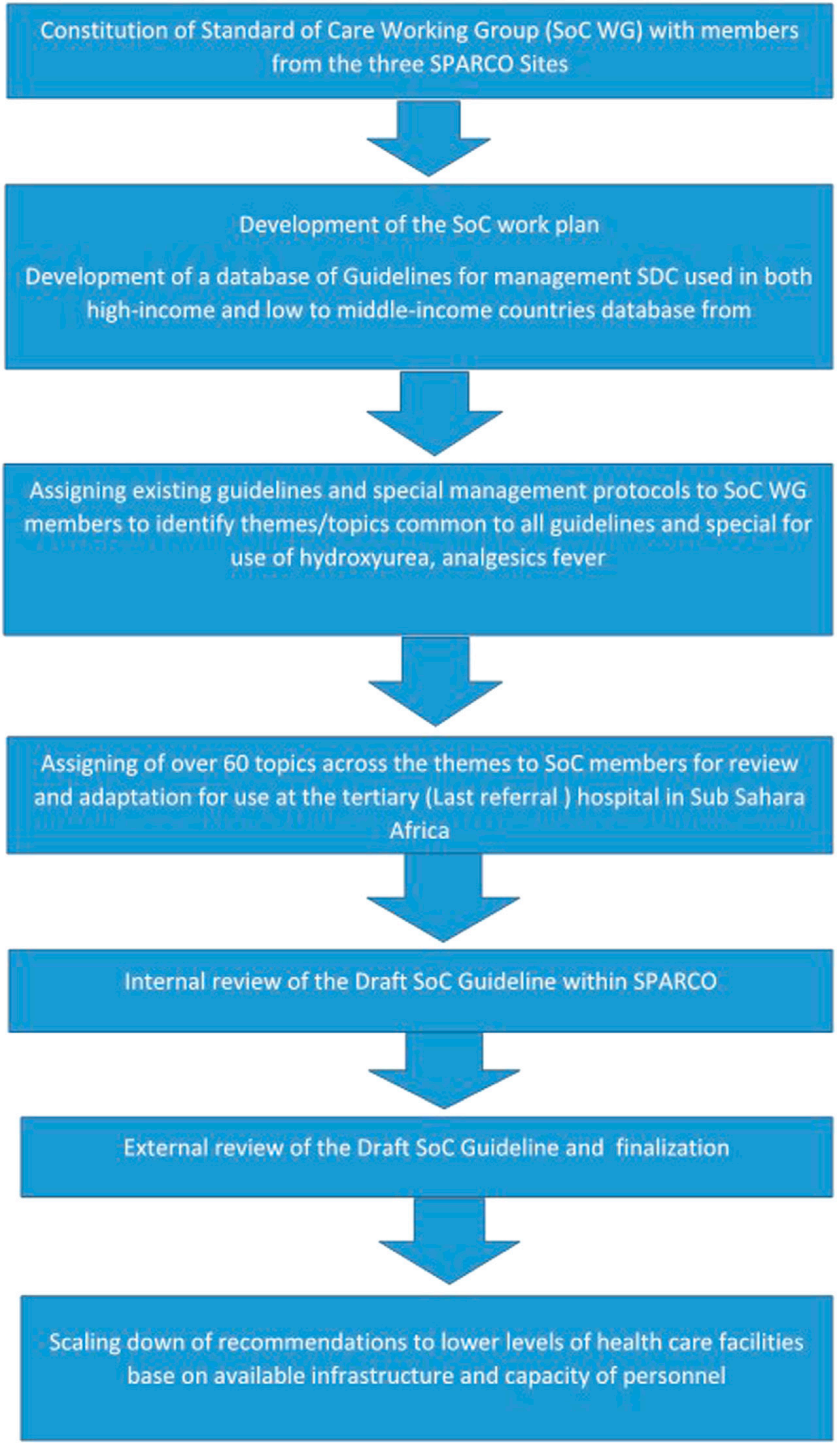
Graphical presentation of the process of development of the standard of care guidelines.

Based on the review of the existing guidelines, the SPARCo WG developed guidelines for the management of SCD in SSA and set minimum standards for management of SCD aligned to institutional technical and human resources for each level of healthcare facility (see levels summarized in Table 1). In developing common guidelines for management of SCD in sub-Saharan Africa, the group successfully developed the first draft of the tertiary facility guidelines for management of SCD and the guidelines developed included all the thematic areas. The first drafts of the tertiary facility level guidelines also referred to as the standard version of the guidelines were internally reviewed by members of the group, and then by the consortium members before being sent out for external review. In developing the guidelines for all the other lower referral levels ie regional, district, health centres, health posts/dispensaries/CHPs level and home level, the same processes were taken but other members of the consortium from Ghana, Tanzania and Nigeria were co-opted into the working group to complete this final aspect of the work and the multi referral level SCD guidelines were compiled. They were reviewed by our senior haematologists in the group before being submitted for internal review by SPARCO hub to other consortium members and subsequently for external review. The processes for the review of the guidelines and drafting of the recommendations are represented in Figure 1.

## Results

The results from this review yielded a guidelines document which had recommendations for management of SCD based on the institutional and human capacity. We developed recommendations for five different levels of healthcare and a sixth level being what has to be done in the home before patients were taken to the hospital. A total of 64 topics had recommendations written out for them (Table 4). The major themes and topics that had recommendations are as seen in Table 4. The recommendations showed what management guidelines could be instituted at each referral level with the option of referring a patient to another referral level health facility for specialist expertise. The home level guidelines focussed on what action was needed in the event of any complication at home and when the patient needed to go for screening for complications of SCD.

Tables 5, 6 are two examples out of the 64 guidelines written. These two illustrate the different recommendations for the screening of stroke and retinopathy in different levels of healthcare facilities and also in the house.

**TABLE 5:** Screening for stroke risk. Transcranial Doppler ultrasonography (TCD) for children at different levels of healthcare facilities.

Last referral hospital
Obtain TCD at least once annually in those with SCD-SS or SCD-Sβ^0^. If TCD is not available at your facility, arrange for the child to visit a centre where it can be performed, or arrange for portable TCD screening to be performed for the child at your facility on a periodic basis
**Level I Health Centre (HC I)**
Refer children with SCD-SS or SCD-Sβ^0^ to the secondary referral hospital (regional centre) for SCD annually for comprehensive evaluation including TCD at least once a year
**First level of care (Home)**
If your child has SCD-SS or SCD-Sβ^0^, ask your doctor or nurse about a test called “TCD”. This test detects those children who may be in danger of having a stroke

**TABLE 6:** Screening for retinopathy SCD disease patients for different healthcare levels.

Last referral hospital
Refer a person with SCD to an ophthalmologist for a dilated eye examination to evaluate for retinopathy beginning at age 10 years. If dilated retinal examination is normal, re-screen at 1–2-year intervals
**Secondary Referral Hospital**
Refer to an ophthalmologist for a dilated eye examination to evaluate for retinopathy beginning at age 10 years. If dilated retinal examination is normal, re-screen at 1–2-year intervals
**Level II Health Centre**
Refer to an accredited centre for dilated eye examination by ophthalmologist to evaluate for retinopathy, as part of Annual Review beginning at age 10 years
**Level I Health Centre**
Refer to an accredited centre for dilated eye examination by ophthalmologist to evaluate for retinopathy, as part of Annual Review beginning at age 10 years
**First level of care (Home)**
From age 10years and above: Ask the doctor or nurse about referral to a retina specialist eye doctor to check the eyes regularly. If the person with SCD complains about his sight or has pain in the eye, she or he should be seen by specialist eye doctor as quickly as possible

Each of the recommendations were different for the different levels of healthcare for screening of stroke (Table 5). For home-based care, the caregiver was advised to ask about the TranscranialDoppler (TCD) ultrasound screening test so that it could be written out for them by the healthcare practitioner. At the level 1 facility which is the Health posts or CHPs compound and Level 11 referral centre which is the health centre, healthcare workers (HCW) are required to refer to the tertiary centre or regional centres where TCD facilities are commonly available. The last referral hospital which are tertiary facilities mostly have TCD facilities available and can perform TCD for children 2–16 years annually.

In Table 6, the primary physician/haematologist are prompted to let the ophthalmologist do a fundoscopy for patients from age 10 at the tertiary level. In the regional hospitals where ophthalmologists are available, patients can be referred to them at the regional facility or to the tertiary hospitals for fundoscopy. Healthcare facilities at lower referral levels are recommended to refer to either the regional or tertiary facilities whilst the home guidelines will alert the parent or patient to ask about the ophthalmology review.

## Discussion

It is essential that every patient with SCD receives comprehensive care that is coordinated at each level by medical experts. The availability of treatment guidelines normally leads to a harmonised way of treating patients. The existing guidelines are commonly developed for the last referral levels or tertiary facility making adaption for use by the primary healthcare very difficult. This can lead to misuse of these available guidelines. The compilation of this document brought to the forefront a harmonised way of treatment of SCD in SSA based on our resources and constraints. The lowest level of comprehensive care for patients with sickle cell disease is available at the healthposts/dispenasaries/CHPs compounds which are mainly manned by nurses and with available recommendations, they will know what can be done at their end and when to refer.

Patients can access care at any of the other lower referral healthcare facilities and be managed with recommendations that are in keeping with institutional resources at that level. When such patients need care that requires expertise that is not available at that level, the recommendations will be to refer to the appropriate referral level where those expertise are available (mainly a higher referral level facility like the regional or tertiary facility). This encourages patients to have good clinical care nearer their homes but also having access to specialist screening modalities and expertise at the tertiary hospitals if need be. With this, patients are not limited to a specific referral level when interventions cannot be instituted for them at that level of healthcare.

One of the lessons learnt during this process was the benefits of task sharing and teamwork. The SoC WG worked as a team with every member carrying out his or her writing assignment dutifully. Another benefit derived from the exercise was the mentorship from the SoC WG Chair, Professor Kwaku Ohene-Frempong to the younger members of the team. It is also noteworthy that with little or no financial resources the SoC WG was able to put together this valuable SoC document which if implemented has potential to improve the care and management of SCD in sub-Saharan Africa.

It is unique in terms of the availability of recommendations for different levels of care as compared to the traditional guidelines which is more focussed at the tertiary levels. With the traditional guidelines, those in the primary healthcare settings find it difficult adapting it for their use.

## Limitations

Time constraint was a challenge for both writers and reviewers as most members are very busy in other equally important duties such as clinical work and teaching at their respective institutions thus often missing timelines and delaying the process though not compromising the quality of work.

## Conclusion and recommendations

This SoC recommendations for SCD is a useful material that can be used for consistent standards of treatment in SSA. We will need to create awareness and advocate for adoption and use among governments and stakeholders such as Paediatricians and Haematologists as well as other specialties involved in managing the complications of SCD.

We recommend the training of stakeholders across medical disciplines, professions and cadres on the use of the guideline and possibly incorporating it in the training curricular of nursing schools, community health workers’ schools and other allied health workers. We will also recommend the distribution of both online/mobile App and both full and short (pocket) versions of the SoC recommendations for SCD and adapted in a local context for implementation in all SSA countries.

## Data Availability

The original contributions presented in the study are included in the article/supplementary material, further inquiries can be directed to the corresponding author.
